# The relationship between pain, disability, guilt and acceptance in low back pain: a mediation analysis

**DOI:** 10.1007/s10865-017-9826-2

**Published:** 2017-02-02

**Authors:** Danijela Serbic, Tamar Pincus

**Affiliations:** Department of Psychology, Royal Holloway, University of London, Egham Hill, Egham, Surrey TW20 0EX UK

**Keywords:** Low back pain, Pain-related guilt, Acceptance, Pain, Disability, Mediation analysis

## Abstract

Pain-related guilt is a common yet unexplored psychological factor in low back pain (LBP). It has recently been linked to greater depression, anxiety and disability in LBP, hence an understanding of how it can be managed in the presence of pain and disability is necessary. Since acceptance of pain has been shown to be associated with improved outcomes in chronic pain, we examined whether it might also help reduce guilt in people with LBP. To this end, a series of mediation analyses were conducted on data from 287 patients with chronic LBP, in which acceptance of pain was tested as a mediator of the relationship between pain/disability and guilt. Results showed that acceptance of pain reduced the impact of pain/disability on pain-related guilt in all mediation analyses. Pain-related guilt might be a potential target for acceptance based interventions, thus this relationship should be further tested using longitudinal designs.

## Introduction

Low back pain (LBP) is the leading cause of daily disability in the world (Lim et al. 2012). It is a debilitating, but also emotionally distressing condition. There is a strong body of evidence showing that psychological factors such as depression, anxiety and fear of pain are related to increased pain and disability in LBP (Linton and Shaw 2011; Pincus and McCracken 2013). One less known psychological factor, which has only recently been studied in the context of LBP is pain-related guilt. Guilt is a moral emotion that refers to a perception that we might have hurt somebody or that we might have done something wrong (Kubany and Watson 2003). It is also a self-conscious emotion, meaning that it is linked to self-identity and how we perceive and value the self (Tangney et al. 2007). Prolonged exposure to chronic pain can potentially result in a defeated self (Tang et al. 2007; Turner-Cobb et al. 2015). For example, recent findings suggest that people with chronic pain report greater levels of mental defeat in comparison to pain-free controls (Turner-Cobb et al. 2015). Additionally, significantly greater levels of guilt were found in people with chronic pain than in pain-free controls. However, this study measured guilt-proneness in general, rather than guilt related to participants’ pain experiences.

Research by Serbic and Pincus (2013, 2014) focused specifically on developing a framework and measurement for pain-related guilt. While all guilt is inherently social, in that it relates to internalised notions of right and wrong, learnt from others, this new model of guilt divides pain-related guilt into three specific components: *social guilt*, which focuses on being unable to participate in social activities and on letting down family and friends; *managing condition/pain guilt*, which is about being unable to manage the pain better; and *verification of pain guilt*, which refers to guilt associated with legitimization of the pain in the absence of concrete evidence and diagnosis. They found that pain-related guilt is a common experience among patients with LBP. There is also evidence that pain-related guilt is associated with pain, disability and mood in LBP (Serbic and Pincus 2014; Serbic et al. 2016). In a recent study with 413 patients with chronic LBP and employing path analysis, it was found that pain-related guilt was associated with diagnostic uncertainty, depression, anxiety and disability in LBP (Serbic et al. 2016). *Social guilt* emerged as the most prominent and troubling type of guilt in this study, and was positively and highly associated with disability, both through and independently of depression. This perhaps is not surprising considering that pain and disability often lead to avoidant behaviours, social withdrawal and worry about how social disengagement might affect others (Newton-John and Williams 2006; Serbic and Pincus 2013; Snelgrove et al. 2013).

Pain-related guilt appears to be a common and unhelpful experience in LBP, thus research should focus on helping patients accept their emotions and cognitions, while striving to live a full life amongst the limitations imposed by pain. The main aim of this study was to understand if acceptance of pain is one such factor. Acceptance of pain is defined as a process of adapting behaviour responses so they are directed towards engaging in activities in spite of pain, rather than avoiding them or focusing on controlling pain (McCracken and Vowles 2006). Acceptance is a component of the Acceptance and Commitment Therapy (ACT) (Hayes et al. 1999). There is a large body of research showing that it leads to improved emotional and physical functioning in people with chronic pain. Greater acceptance of pain has been found to be associated with better psychological wellbeing and less pain, disability and distress (McCracken 1998; McCracken et al. 2004a, b; Viane et al. 2003). Specifically, in one study it was demonstrated that higher levels of acceptance of pain in 128 people with chronic pain were associated with adaptive responses to pain, independent of the influences of depression and pain intensity (McCracken et al. 1999). Furthermore, McCracken and Gutiérrez-Martínez (2011) found that increases in acceptance of pain significantly correlated with decreases in disability, anxiety and depression, at a three-month follow-up, independent of changes in pain. These findings suggest that acceptance can be used to address a range of outcomes in people with chronic pain (McCracken and Vowles 2014). A recent meta-analysis found that acceptance strategies are useful emotion- regulation strategies in treatments for chronic pain, and they are more beneficial than other emotion regulation strategies, such as distraction and reappraisal with respect to pain tolerance (Kohl et al. 2012).

The rationale for this study is based on the notion that patients are caught between feeling that they ought to behave in a certain way and be different, but cannot be this desired person because of their pain. It is logical that acceptance of their pain and their self will reduce the negative feelings, such as guilt, which emerge from the dissonance of unchangeable reality and unrealistic expectations. With regards to specific types of pain-related guilt, patients who experience *social guilt* are also more likely to engage in social withdrawal and avoidance behaviours (Serbic and Pincus 2013). However, the causal path between social guilt and avoidance behaviours is unknown; it could also be that avoidance behaviours initiate social guilt, which might lead to further avoidance of social engagement, and creating a vicious cycle. In chronic pain patients, greater acceptance of pain has been associated with less avoidance (de Boer et al. 2014; McCracken 1998), thus greater acceptance of pain may also be associated with reduced *social guilt*. Acceptance of pain, and indeed, acceptance in general, is a process of increasing psychological flexibility, thus engaging fully with life, while accepting features that are beyond control and cannot be changed (McCracken and Vowles 2014). Guilt related to management and verification of pain is often related to aspects of pain that are beyond patients’ control, e.g. feeling guilty about not having a clear diagnosis and/or not being able to control the pain. Therefore, greater acceptance of pain may potentially reduce feelings of guilt related to both *management of condition/pain* and *verification of pain*.

All this suggests that people who have high levels of pain-acceptance and psychological flexibility may report lower levels of guilt, even in the presence of high pain and disability. To examine this, we planned to employ mediation analysis. Mediation tests if the relationship between a predictor and an outcome variable can be explained by their relationship to a third, mediator variable (Field 2013). It was predicted that acceptance of pain would be a mediator of the relationship between pain and *social guilt*, verification of pain guilt and managing condition/pain guilt. It was also predicted that acceptance of pain would mediate the relationship between disability and these three types of pain-related guilt.

## Method

### Participants

This was a subsample from a larger study who provided acceptance of pain ratings. Inclusion criteria were that participants be over the age of 18 years and have chronic (>3 months) musculoskeletal LBP. Participants with back pain due to ankylosing spondylitis, osteoporosis, cancer and inflammatory conditions such as rheumatoid arthritis were excluded. A total of 295 participants who met inclusion criteria were included: 136 participants were recruited from two pain clinics and a physiotherapy department from the London National Health Service (NHS); 159 participants were recruited from a clinic of osteopathy in London. For participants recruited in NHS the inclusion criteria were checked by clinician in participating clinics. For participants recruited in the clinic of osteopathy the inclusion criteria was established by self-report. Participants who were missing more than 10% of responses on any of the scales were excluded from the analysis (N = 8) (Bennett 2001). The total sample size for this analysis was 287. Ethical approval was obtained from the university research ethics committee, National Health Service (NHS), and participating institutions.

## Materials

The following measures were included:

*Pain*-*related guilt*—The pain-related guilt scale (PGS) was developed in a series of mixed-methods studies (Serbic and Pincus 2013). It includes 12 items which form three subscales representing three types of guilt in LBP: *social guilt* (4 items), which relates to letting down family and friends; *managing condition/pain guilt* (5 items), which is about being unable to control pain; and *verification of pain guilt* (3 items), which relates to the lack of diagnosis and evidence for the pain. Responses are on a Likert-type rating scale: 1 = ‘never’ feeling guilty to 5 = ‘always’ feeling guilty. Preliminary validations of the PGS showed that the three subscales had good validity and reliability (Serbic and Pincus 2014).

*Acceptance of pain*—The Chronic Pain Acceptance Questionnaire (CPAQ) (McCracken et al. 2004a, b) is a widely used measure of pain acceptance. A shortened form of the CPAQ, CPAQ-8 was developed (Fish et al. 2010) and used in this study in order to reduce response burden. It consists of eight items, with total scores ranging from 0 to 48. The 8 items are rated on a 7-point scale and form two subscales (4 items in each): *activity engagement*, which is about engaging in life activities with pain present; and *pain willingness*, which relates to a relative absence of attempts to control pain. Validations of the scale suggest good validity and reliability, similar psychometric properties to the CPAQ (Fish et al. 2010) and sensitivity to rehabilitation changes (Rovner et al. 2014).

*Anxiety and Depression*—The Hospital Anxiety and Depression Scale (HADS) (Zigmond and Snaith 1983) is a widely used screening measure of anxiety and depression in medical populations. It consists of 7 anxiety and 7 depression items. Scores range from 0 to 21 for each scale and higher scores indicate greater likelihood of depression or anxiety. Recommended cut-offs are: 8–10: mild cases, 11–15: moderate cases and 16 or above: severe cases (Zigmond and Snaith 1983).

*Disability*—Roland Disability Questionnaire (RDQ) (Roland and Morris 1983) is a widely used and reliable measure of low back disability (Waddell 2004). It consist of 24 yes/no questions where 0 = no disability to 24 = maximum disability.

*Demographics and pain details*—Participants were asked to give details about their age, gender, duration of their back pain (0–3, 3–6, 7–12 months, 1–2, 2–3, 4–5, 5+, 10+ years) and pain intensity. Pain intensity was measured using a single question: ‘How would you rate your back pain over the past week on a scale of 0—10, where 0 is ‘no pain’ and 10 is ‘pain as bad as could be’? (Cleeland and Ryan 1994).

### Study design and planned analyses

This was a cross sectional design. A series of correlational and regression mediation analyses were planned. Firstly, we wanted to establish if the two key constructs, pain-related guilt and acceptance of pain were correlated. To this end, zero order correlations were planned between the PGS and CPAQ-8 scale, as well as between their subscales. As the total acceptance of pain score (CPAQ-8) was tested as a mediator in the mediation analyses it was necessary to examine if it was correlated with its two subscales, *pain willingness* and *activity engagement*.

**Fig. 1:**
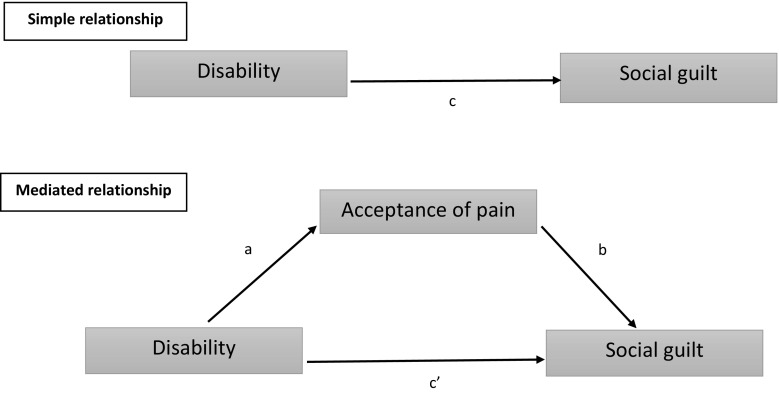
Diagram of an example mediation model *c*—total effect of predictor on outcome without the mediator in the model; *c’*—direct effect of predictor on outcome while controlling for the mediator; *a*—effect of the predictor on the mediator; *b*—effect of the mediator on the outcome; *ab*—indirect effect of predictor on outcome thorough the mediator

In our regression mediation models, pain intensity and disability were planned to be examined as predictors of the three types of pain-related guilt. Furthermore, it was planned to examine if this relationship might be mediated by acceptance of pain. Figure 1 shows an example mediation model.

All mediation analyses were performed using PROCESS (Hayes 2013). PROCESS is based on regression-based path-analytic framework and estimates the indirect effect and bias-corrected confidence intervals (CI). An indirect effect is considered significant when the CI do not include zero (Field 2013). Additionally, the level of this significance was assessed using Sobel tests. All analyses were based on 1000 bootstrapping samples. Bootstrapping is a nonparametric resampling procedure, and as such, it does not violate assumptions of normality. The size of the indirect (mediation) effect was expressed with kappa squared (*k* ^2^): small effect is .01, a medium effect is around .09, and a large effect is around .25 (Preacher and Kelley 2011). Bootstrapped confidence intervals were presented for all mediation effects (Field 2013).

Prior to the main analyses, descriptive statistics were reported for all variables in the study, as well as *t* tests ad Chi square tests to examine baseline sex differences. These analyses were performed using SPSS version 21.0 (IBM 2013).

## Results

### Descriptive statistics

See Table 1 for descriptive statistics. There were no significant differences between male and female participants on any measured variables.

**Table 1:** Sample characteristics

	Male N = 97	Female N = 190	Inferential statistics	Effect size	Total N = 287
	Mean/SD or %	Mean/SD or %	t/χ^2b^	d/odds ratio	Mean/SD or %
Age^a^	50.89 (15.8)	49.38 (15.14)	.78	.10	49.88 (15.35)
Pain duration^a^ >12 months (%)	87.8	90.8	.67	1.38	89.5
Pain intensity	6.19 (2.35)	6.31 (2.39)	.40	.05	6.26 (2.37)
Depression	7.49 (4.55)	7.33 (3.98)	.31	.04	7.39 (4.17)
Anxiety	9.10 (4.49)	9.46 (4.34)	.65	.08	9.34 (4.38)
Disability	11.86 (6.88)	10.81 (5.99)	1.28	.16	11.16 (6.31)
Verification of pain guilt	2.74 (1.24)	2.69 (1.29)	.28	.04	2.71 (1.27)
Social guilt	2.92 (1.33)	2.89 (1.24)	.15	.02	2.90 (1.27)
Managing condition/pain guilt	2.99 (1.21)	2.91 (1.24)	.56	.07	2.93 (1.23)
Total acceptance of pain (CPAQ-8)	23.96 (9.75)	24.12 (7.8)	.14	.02	24.1 (8.49)
Pain willingness	8.95 (5.31)	8.32 (4.76)	1.02	.13	8.53 (4.95)
Activity engagement	15.01 (6.29)	15.79 (5.70)	1.06	.13	15.53 (5.91)

^a^N (sample size) was different for age (N = 283, 94 males) and pain duration (N = 286, 96 males); ^b^t—t test, χ^2^—Chi Square; no significant differences were found between male and female participants on any variables

### Correlations

Table 2 shows zero order correlations between all variables examined in the regression mediation models. The correlations between pain-related guilt (PGS) and acceptance of pain (CPAQ-8), and their subscales were all negative and significant, indicating that the two constructs were related. The correlations between the total acceptance of pain sore and its two subscales (*pain willingness* and *activity engagement*) scores were large, positive and highly significant, therefore it was plausible to use the total acceptance score in further mediation analyses.

**Table 2:** Zero order correlations between variables examined in the regression mediation models

	Pain intensity	Disability	Total acceptance (CPAQ-8)	Pain willingness	Activity engagement	Overall guilt (PGS)	Verification of pain guilt	Social guilt
Disability	.60							
Total acceptance (CPAQ-8)	−.42	−.65						
Pain willingness	−.37	−.47	.73					
Activity engagement	−.29	−.53	.82	.22				
Overall guilt (PGS)	.48	.56	−.59	−.45	−.40			
Verification of pain guilt	.38	.36	−.39	−.26	−.35	.86		
Social guilt	.44	.63	−.68	−.53	−.54	.89	.62	
Managing condition/pain guilt	.47	.49	−.51	−.40	−.40	.95	.78	.75

Pearson correlations are reported, all two tailed and significant at *p* < .001

### Reliability analysis

Cronbach alpha value for the acceptance of pain scale (CPAQ-8) was .76, suggesting good reliability. Its subscales showed adequate (*pain willingness* = .68) to good reliability (*activity engagement* = .88). Pain-related guilt scale (PGS) showed excellent reliability (.95). Cronbach alpha values for its subscales were either good or excellent: *social guilt* = .93, *managing condition/pain guilt* = .92 and *verification of pain guilt* = .88.

### Main analysis

The assumption of multicollinearity was met, all correlations were between +.9 and −.9 (see Table 2). See Table 3 for all mediation analyses results.

**Table 3:** Outcomes of mediation analyses from pain/disability to the three types of pain-related guilt assessing indirect effects of acceptance of pain

N = 287	Models without mediator		Models with mediator									
		B	B					Bootstrap results for indirect effects (95% CI)		Bootstrap results for indirect effect sizes (95% CI)		
	*R* ^2^	c	*R* ^2^	c’	a	b	ab	Lower	Upper	*k* ^2^	Lower	Upper
Pain-Acceptance-Guilt												
Pain-Acceptance-VG	.142***	.202***	.208***	.138***	−1.49***	−.043***	.063***	.039	.097	.116	.070	.172
Pain-Acceptance-SG	.195***	.237***	.498***	.102***	−1.49***	−.091***	.135***	.099	.173	.267	.202	.329
Pain-Acceptance-MG	.219***	.242***	.337***	.160***	−1.49***	−.055***	.082***	.054	.115	.162	.116	.229
Disability-Acceptance-Guilt												
Disability-Acceptance-VG	.127***	.072***	.172***	.036*	−.868***	−.041***	.036***	.019	.055	.146	.076	.219
Disability-Acceptance-SG	.400***	.127***	.530***	.066***	−.868***	−.071***	.061***	.047	.078	.301	.232	.365
Disability-Acceptance-MG	.241***	.095***	.303***	.054***	−.868***	−.047***	.041***	.025	.056	.185	.117	.257

*VG* verification of pain guilt; *SG* social guilt; *MG* managing condition/pain guilt; *B* unstandardized regression weight; *c* total effect of predictor on outcome without the mediator in the model; *c’* direct effect of predictor on outcome while controlling for the mediator; *a* effect of the predictor on the mediator; *b* effect of the mediator on the outcome; ab indirect effect of predictor on outcome thorough the mediator; *R* ^2^ amount of variance explained by the model; *CI* confidence intervals; *k* ^2^ effect size

* Significant at *p*  <  .05; ** significant at *p*  <  .01; *** significant at *p*  <  .001

#### Pain–Acceptance–Guilt mediation analyses

There was a significant indirect (mediation) effect of pan intensity through acceptance of pain on *verification of pain guilt* (medium effect size), *social guilt* (large effect size) and *managing condition/pain guilt* (medium effect size). Acceptance of pain significantly reduced the impact of pain intensity in all three analyses when compared to the regression models without the mediator (See Table 3 for all regression mediation results).

#### Disability–Acceptance–Guilt mediation analyses

There was a significant indirect effect of pain intensity through acceptance of pain on *verification of pain guilt* (medium effect size), *social guilt* (large effect size) and *managing condition/pain guilt* (medium effect size). Acceptance of pain significantly reduced the impact of disability in all three analyses when compared to the models without the mediator (See Table 3).

## Discussion

### Main findings and fit with past research

The results show that acceptance of pain mediated the relationship between pain and the three types of pain-related guilt, and also the relationship between disability and the three types of pain-related guilt. Acceptance of pain reduced the impact of pain intensity and disability in all mediation analyses and the largest effect sizes were found with *social guilt*.

Our findings support findings from other studies in which the mediating role of acceptance of pain was also found (Akerblom et al. 2015; Fish et al. 2010). For example, mediating effects of acceptance of pain were also found in Fish et al.’s (2010) study in which acceptance of pain (also measured with CPAQ-8) partly meditated the relationship between pain intensity and pain interference, depression and anxiety (also measured with HADS) in 428 people with chronic pain. Thus, acceptance of pain can reduce the impact of pain severity on patients’ functioning and emotional distress, including pain-related guilt.

An important question is, how might acceptance of pain regulate the impact of pain and disability on pain-related guilt? Research on guilt (in non-pain contexts) has shown that there are several ways of dealing with guilt. Guilt can stimulate constructive and proactive responses, for instance a person who has transgressed may try to repair the negative consequences, or at least, they can apologise. And, when external amends are not possible, they can try to avoid guilt-evoking behaviours in the future (Tangney et al. 2007). But, pain and disability are likely to prevent patients with back pain from responding in such ways. A major theme throughout pain-related guilt is inability to control not only the pain experience itself, but the process of ‘rising above it’. Patients report not only feeling guilty about behaviours that result from the pain experience, which limit their roles and activities, but feeling guilty about failing to recover, and indeed, experiencing the pain itself. This may result in problematic guilt responses such as: (a) Withdrawal from social situations: for example, patients who experience *social guilt* may find it difficult to make amends by being more socially proactive. They might be more likely to engage in avoidant behaviours instead. (b) Developing or reinforcing a biased sense of responsibility: for example, patients who experience guilt related to management and verification of pain might continue to search for ways of verifying and controlling aspects of pain that are beyond their control. (c) Emotional distress: feelings of guilt and aforementioned guilt responses are likely to result in emotional distress. This is supported by research which showed that pain-related guilt is linked to increased anxiety and depression in LBP (Serbic et al. 2016). Therefore, addressing the ‘beyond control’ aspect of chronic pain seems important for enabling patients to deal with guilt effectively.

Research suggests that acceptance of one’s pain and condition is one way of addressing the ‘beyond control’ aspect of chronic pain (McCracken and Vowles 2014). For instance, patients who are more acceptant of their pain are also more able to open up to experiences that are beyond their control and do not struggle with them as much as those patients who are less accepting. Furthermore, they might also be able to engage in activities while in the presence of these ‘beyond their control’ experiences. Thus, being more acceptant of pain might result in fewer avoidant behaviours, being less worried about aspects that are beyond one’s control, greater social engagement, and as suggested by our findings, in reduced pain-related guilt.

In summary, there are at least two possible pathways which should be explored by future research: (a) Acceptance reduces avoidance behaviours and increases activity which in turn may result in less guilt, because there is less to feel guilty about. (b) While the process of acceptance does not aim to reduce guilt, but rather involves learning to live fully amongst all the various emotional and cognitive reactions to the experience of pain, research suggests that over time increases in acceptance are associated with reductions in negative emotions such as anxiety and depression, and potentially also guilt.

Weakened social roles and reduced social contacts are common problems in LBP (Harris et al. 2003; Snelgrove et al. 2013). *Social guilt* is common and probably the most important type of guilt needing to change because it is closely linked to disability (Serbic et al. 2016). In the current study, mediation effects of acceptance of pain were most prominent with *social guilt*, which is a promising finding. Possibly this supports the first pathway above rather than the second: it is through increased activity with others that guilt is reduced, but this needs to be tested explicitly in future research using prospective designs and measures of social engagement. The purpose of the current study was to conduct an initial exploration of the relationship between guilt, acceptance and pain/disability; future research should test more complex models using longitudinal designs.

## Implications and future research

Interventions such as ACT, Contextual Cognitive Behavioural Therapy and Mindfulness all aim to help people increase their psychological flexibility. This in turn allows people to live their life to the full amongst the unavoidable challenges posed by life, including pain. Considering the mediating role of acceptance of pain in the relationship between pain/disability and pain-related guilt in the current study, future research could examine whether interventions that target to increase acceptance and psychological flexibility also result in reduced pain-related guilt. This seems plausible because acceptance and mindfulness-based interventions address a range of feelings such as anger, anxiety, depression and fear (McCracken and Vowles 2014). For example, a meta-analysis of acceptance and mindfulness-based treatments, which included 22 studies and 1235 patients with chronic pain, and which used pain intensity and depression as primary measures, and anxiety, physical wellbeing, and quality of life as secondary measures found that these treatments are as effective as CBT (Veehof et al. 2011).

Furthermore, understanding specific patients’ needs and identifying targets for interventions is important for improving outcomes in LBP (Pincus and McCracken 2013). Recent research suggests that guilt may be a risk factor for poor outcome (Serbic et al. 2016), and the current study findings suggest that it might be a promising target for acceptance based interventions. Therefore, our findings support the argument that targeting pain-related guilt explicitly could potentially improve pain management treatments.

## Limitations

This research is in early stages and there is a need to be cautious about interpretation of the findings. The study is cross sectional, therefore the direction of the relationship between the studied variables cannot be inferred. Prospective research should be employed in future to examine the direction of relationships. The mediation models tested are rather simple and limited; there could be other mediating and moderating factors affecting the relationship between pain/disability and pain-related guilt. The sample used in this study may not represent broader LBP patient populations within or outside of the UK, thus our findings may not be entirely applicable in non-western cultures. Additionally, there is evidence suggesting that guilt may be qualitatively different across different cultures (Bedford and Hwang 2003). As in all self-report measures, there is a threat of social-desirability bias (Stangor 1998), therefore self-reported feelings of guilt may not accurately represent actual feelings.

## Conclusions

To our knowledge this is the first study to examine the relationship between pain-related guilt and acceptance of pain in LBP, therefore these findings should be retested and replicated in new samples. Although the current mediation models may appear to be restrictive, they provide initial evidence for the role of acceptance in pain-related guilt. Taken together, these findings suggest that acceptance of pain affects the relationship between pain/disability and pain-related guilt, and its role is in particular prominent in *social guilt*. This relationship should be further tested using longitudinal designs.
